# The dilemma of neuroprotection trials in times of successful endovascular recanalization

**DOI:** 10.3389/fneur.2024.1383494

**Published:** 2024-04-09

**Authors:** Antje Schmidt-Pogoda, Johannes Kaesmacher, Nadine Bonberg, Nils Werring, Jan-Kolja Strecker, Mailin Hannah Marie Koecke, Carolin Beuker, Jan Gralla, Raphael Meier, Heinz Wiendl, Heike Minnerup, Urs Fischer, Jens Minnerup

**Affiliations:** ^1^Department of Neurology with Institute of Translational Neurology, University of Münster, Münster, Germany; ^2^University Institute of Diagnostic and Interventional Neuroradiology, University Hospital Bern, Inselspital, University of Bern, Bern, Switzerland; ^3^Institute of Epidemiology and Social Medicine, University of Münster, Münster, Germany; ^4^Department of Neurology, University Hospital Basel, University of Basel, Basel, Switzerland; ^5^Department of Neurology, University Hospital Bern, University of Bern, Bern, Switzerland

**Keywords:** acute stroke, neuroprotection, mechanical thrombectomy, translational failures, infarct growth

## Abstract

**Background:**

The “translational roadblock” between successful animal stroke studies and neutral clinical trials is usually attributed to conceptual weaknesses. However, we hypothesized that rodent studies cannot inform the human disease due to intrinsic pathophysiological differences between rodents and humans., i.e., differences in infarct evolution.

**Methods:**

To verify our hypothesis, we employed a mixed study design and compared findings from meta-analyses of animal studies and a retrospective clinical cohort study. For animal data, we systematically searched pubmed to identify all rodent studies, in which stroke was induced by MCAO and at least two sequential MRI scans were performed for infarct volume assessment within the first two days. For clinical data, we included 107 consecutive stroke patients with large artery occlusion, who received MRI scans upon admission and one or two days later.

**Results:**

Our preclinical meta-analyses included 50 studies with 676 animals. Untreated animals had a median post-reperfusion infarct volume growth of 74%. Neuroprotective treatments reduced this infarct volume growth to 23%. A retrospective clinical cohort study showed that stroke patients had a median infarct volume growth of only 2% after successful recanalization. Stroke patients with unsuccessful recanalization, by contrast, experienced a meaningful median infarct growth of 148%.

**Conclusion:**

Our study shows that rodents have a significant post-reperfusion infarct growth, and that this post-reperfusion infarct growth is the target of neuroprotective treatments. Stroke patients with successful recanalization do not have such infarct growth and thus have no target for neuroprotection.

## Introduction

Decades of acute stroke research have come to an unanimous conclusion: “Everything works in animals, but nothing works in people” (1–3). This so called “translational roadblock” between successful animal studies and neutral clinical trials was usually attributed to conceptual weaknesses (4–7). Nonetheless, we hypothesized that intrinsic pathophysiological differences between rodents and humans, i.e., differences in infarct evolution, contribute to translational failures of neuroprotective stroke drugs.

Most animal studies use models of transient middle cerebral artery occlusion (tMCAO). The pharmacological neuroprotective treatment is usually initiated after reperfusion and neuroprotective efficacy is determined by infarct volume assessment (4). This implies that post-reperfusion infarct volume growth is the therapeutic target of neuroprotection in animal studies. Thus, the question arises if and to what extend infarct volume growth is present in human stroke patients. Considering the huge treatment effects of recanalizing therapies, we hypothesized that so called neuroprotective agents can have only limited additional value after complete endovascular recanalization in the majority of patients.

Here, we compare the infarct evolution in rodents with and without neuroprotective treatments with that in human stroke patients with and without successful thrombectomy, and we illustrate why the concept of neuroprotection requires a thorough selection of suitable stroke patients in the clinical setting.

## Methods

### Data sources

For animal data, we systematically searched Pubmed from the beginning until July 2020 using the terms MRI AND stroke AND animal model AND infarct volume or MRI AND stroke AND rodent AND infarct volume or MRI AND stroke AND mouse AND infarct volume or MRI AND stroke AND rat AND infarct volume. For clinical data, we used a large cohort of stroke patients admitted to the University Hospital Bern (Inselspital) between January 2012 and July 2017.

### Study selection and data extraction of animal studies

We included rodent studies that (a) used either transient middle cerebral artery occlusion (tMCAO) or permanent middle cerebral artery occlusion (pMCAO) for stroke induction and (b) provided at least two sequential MRI scans for infarct volume assessment. The first MRI scan had to be after reperfusion but no later than 6 h after stroke onset. The second scan had to be one or two days later. A detailed description of study selection criteria of the systematic review and data extraction is provided in the Supplementary methods. A PRISMA Checklist is also provided in the Supplementary methods.

### Data analysis of animal studies

Delayed infarct volume growth was determined by the change in mean infarct sizes between time point 1 and 2 per study [(mean volume at t2/mean volume at t1) × 100–100%].

### Selection of stroke patients and documentation of clinical findings

We used a cohort of consecutive stroke patients with clinically suspected large vessel occlusion, who received a primary MRI scan upon admission and at least one follow up-scan one or two days later. We decided to use MRI scans upon admission as baseline scans, because MRI immediately after reperfusion is generally scarce. If compared to MRI scans immediately after reperfusion, MRI scans upon admission overestimate the infarct volume growth rather than underestimate it due to potential infarct volume growth between first scan and successful thrombectomy. In our analyses, a TICI 2b or TICI 3 thrombectomy was regarded as a successful recanalization, thus reflecting the condition in animals with tMCAO, while a TICI 0 to TICI 2a thrombectomy was regarded as an unsuccessful recanalization, thus reflecting the condition in animals with pMCAO. We acknowledge the crucial distinction between recanalization and reperfusion; while recanalization refers to the reopening of the occluded vessel, reperfusion denotes the restoration of blood flow to the affected downstream tissue, which may not automatically ensue following successful recanalization due to various pathophysiological factors.

### Data analysis of stroke patients

Infarct evolution in human stroke patients was determined on an individual level by (volume at time point 2/volume at time point 1) × 100–100%. A linear mixed model with random intercepts per patient was conducted to assess the association of time with ln of infarct size. An overall regression line given by the fixed effects of the mixed model is shown in the plot.

## Results

### Characteristics of included studies and study subjects

**Figure 1:**
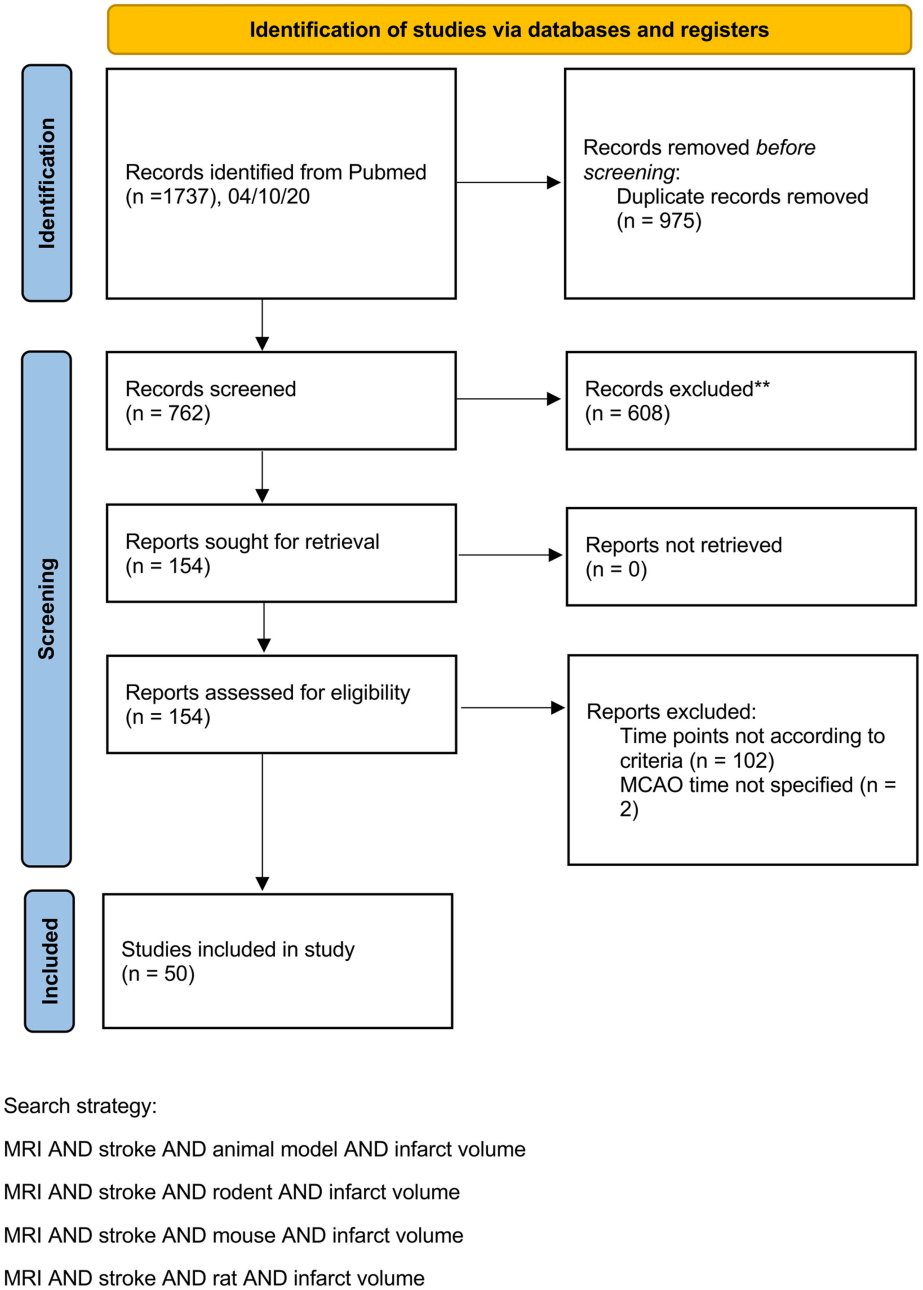
PRISMA flow chart illustrating the identification of animal studies.

Our preclinical meta-analyses included 50 studies from 32 different research groups with 676 animals. A PRISMA flow chart is provided in Figure 1. A table including study details of all studies is provided in the Supplementary Table S1.

Our analysis of infarct growth in stroke patients included 107 patients. The mean age was 69 years, the mean NIHSS upon admission was 10, and the mean duration from symptom onset to groin puncture was 5 h.

### Infarct growth is the target of neuroprotection in animal stroke, but stroke patients with successful recanalization do not offer this target

**Figure 2:**
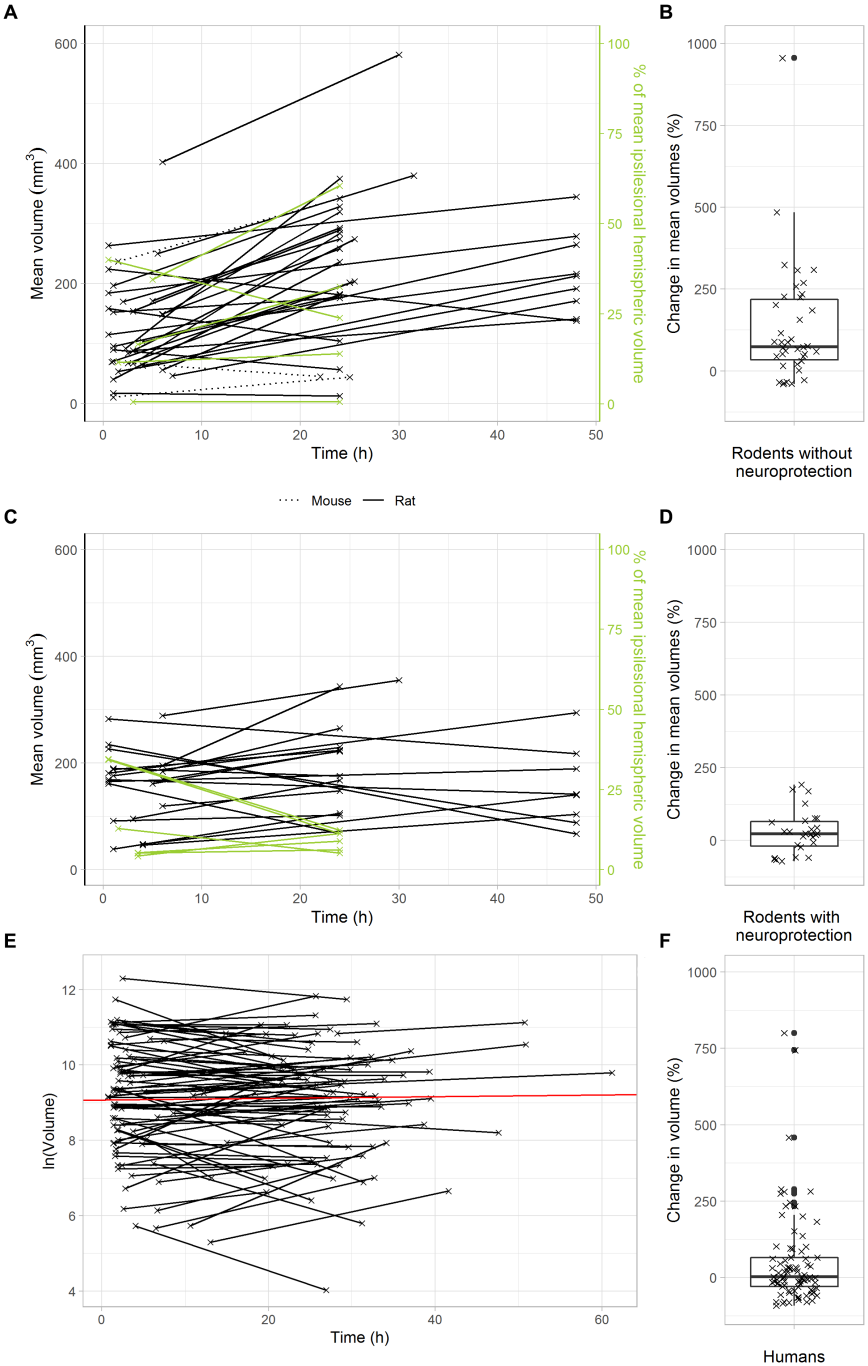
Delayed infarct volume growth is the target of neuroprotection in animal stroke, but most stroke patients with successful recanalization do not offer this target. **(A,B)** Infarct growth after tMCAO in rodents without neuroprotective treatment. **(A)** Black and green lines connect the mean infarct volumes of untreated animals determined at timepoints 1 and 2 in each individual study. **(B)** Each cross represents the change in mean infarct volumes in an individual study. The median change in mean infarct volumes was 74%. **(C,D)** Infarct growth after tMCAO in rodents with neuroprotective treatment. **(C)** Black and green lines connect the mean infarct volumes of treated animals determined at timepoints 1 and 2 in each individual study. **(D)** Each cross represents the change in mean infarct volumes in an individual study. The median change in mean infarct volumes was 23%. **(E,F)** Infarct growth in stroke patients with TICI 2b-3 thrombectomy. **(E)** Black lines connect the infarct volumes at timepoints 1 and 2 in each patient. The red line represents an overall regression line given by the fixed effects of the mixed model. **(B)** Each cross represents the infarct volume growth in a patient. The median change in infarct volumes was only 2%.

To analyze the temporal dynamics of infarct volume progression in animal stroke, we identified stroke studies providing at least two sequential MRI scans, with a first scan within the first six hours after transient middle cerebral artery occlusion and a second scan one or two days later. Among all studies that matched the inclusion criteria as detailed above, 84% reported increasing infarct volumes in untreated animals over the first two days after tMCAO. In untreated animals, the median infarct growth over the first two days after tMCAO was 74% (Figures 2A,B).

We next analyzed the effects of neuroprotective treatments on infarct volume progression in animal stroke. To this end, we searched the above data set for studies, in which the effectiveness of neuroprotective treatments was investigated by sequential MRI scans. As expected, our results confirm a powerful treatment effect of neuroprotectants in animal stroke: In treated animals, there was only a small median infarct growth of 23% in the first two days after stroke (Figures 2C,D). Compared to untreated animals, neuroprotective treatments reduced infarct growth to less than a third. These data clarify that delayed infarct growth is the target of neuroprotective treatments in animal stroke.

Considering that neuroprotective treatments with powerful effects in animals have always failed in large clinical trials, we hypothesized that delayed infarct growth might simply not occur in human stroke patients with transient large vessel occlusion. In other words, stroke patients might just not offer a target for neuroprotection. To verify this hypothesis, we used a large dataset of 119 consecutive stroke patients with clinically suspected large vessel occlusion, who were admitted to the Inselspital Bern and received a primary MRI scan upon admission and at least one follow up-scan one or two days later. Among these 119 patients, 51 patients had a TICI 2b or TICI 3 thrombectomy, indicating almost complete reperfusion. In these patients, the median infarct volume increase was only 2% (Figures 2E,F).

Altogether, these findings confirm our intriguing hypothesis that there is no relevant infarct growth after successful recanalization of large artery stroke in human stroke patients, i.e., these patients have no target for neuroprotection.

### Infarct growth depends on ischemia duration and recanalization success

**Figure 3:**
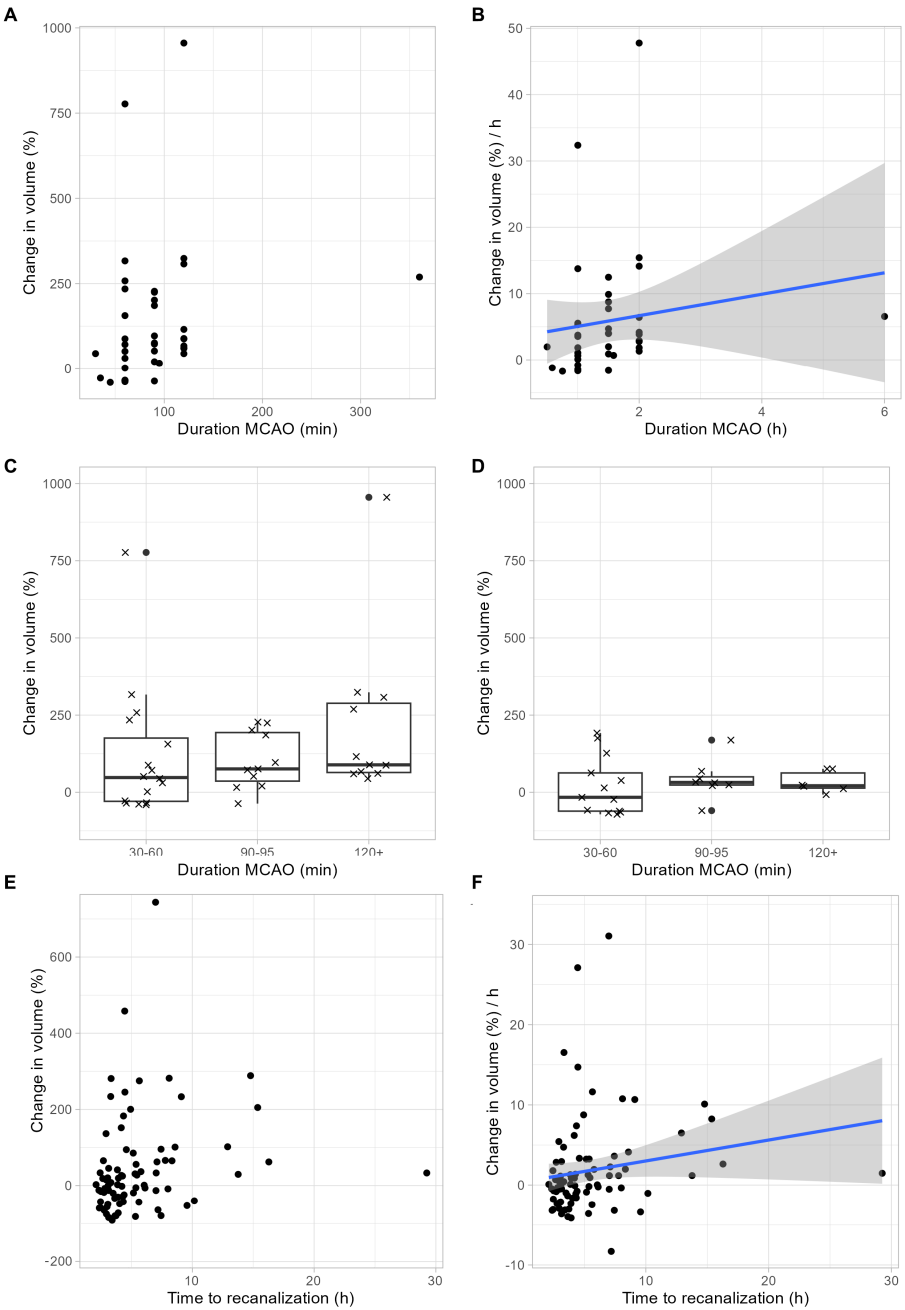
Infarct growth depends on ischemia duration. **(A–C)** Infarct growth after tMCAO in rodents without neuroprotective treatment. **(A)** Infarct growth between MRI 1 and MRI 2 based on the duration of ischemia. Each dot represents the mean infarct growth in an individual study. **(B)** Estimated infarct growth per hour based on the duration of ischemia. Each dot represents an individual study. Assuming a linear growth of infarct volume within the first 48 h, the expected infarct growth per hour can be calculated for ischemia durations between 0 and 2 h using the following formula: 1.62 × t (t: duration of ischemia in hours; t < = 2). This formula is limited to ischemic times between 0 and 2 h. We assume that the curve flattens and is no longer linear with longer ischemic durations, but we cannot estimate this from our available data. **(C)** Each cross represents the change in mean infarct volumes in an individual study. The median change in mean infarct volumes was 48% after 30–60 min ischemia duration, 76% after 90–95 min ischemia duration and 89% after > = 120 min ischemia duration. **(D)** Infarct growth after tMCAO in rodents with neuroprotective treatment. Each cross represents the change in mean infarct volumes in an individual study. The median change in mean infarct volumes was-16% after 30–60 min ischemia duration, 32% after 90–95 min ischemia duration and 21% after > = 120 min ischemia duration. **(E,F)** Infarct growth based on ischemia duration in human stroke patients. **(E)** Infarct growth between MRI 1 and MRI 2 based on the duration of ischemia. Each dot represents the infarct growth of an individual patient. **(F)** Assuming a linear growth of infarct volume within the first 48 h, the expected infarct growth per hour can be calculated for ischemia durations between 0 and 2 h using the following formula: 0.26 × t (t: duration of ischemia in hours; t < = 16). This formula is limited to ischemic times between 0 and 16 h. We assume that the curve flattens and is no longer linear with longer ischemic durations, but we cannot estimate this from our available data.

Next, we examined to which extent infarct growth depends ischemia duration. Our rodent data suggest a linear relationship between infarct growth and ischemia duration (Figures 3A,B). Regardless of the duration of ischemia, rodents benefited from neuroprotective therapy: With a tMCAO duration of 30–60 min, the median infarct volume growth was reduced from 48% to-16%, with a tMCAO duration of 90–95 min, the median infarct volume growth was reduced from 76 to 32%, and with an ischemia duration of > = 120 min, the median infarct volume growth was reduced from 89 to 21% (Figures 3C,D). In accordance with our animal data, our human data also show an increase in infarct growth depending on the duration of ischemia (Figures 3E,F).

**Figure 4:**
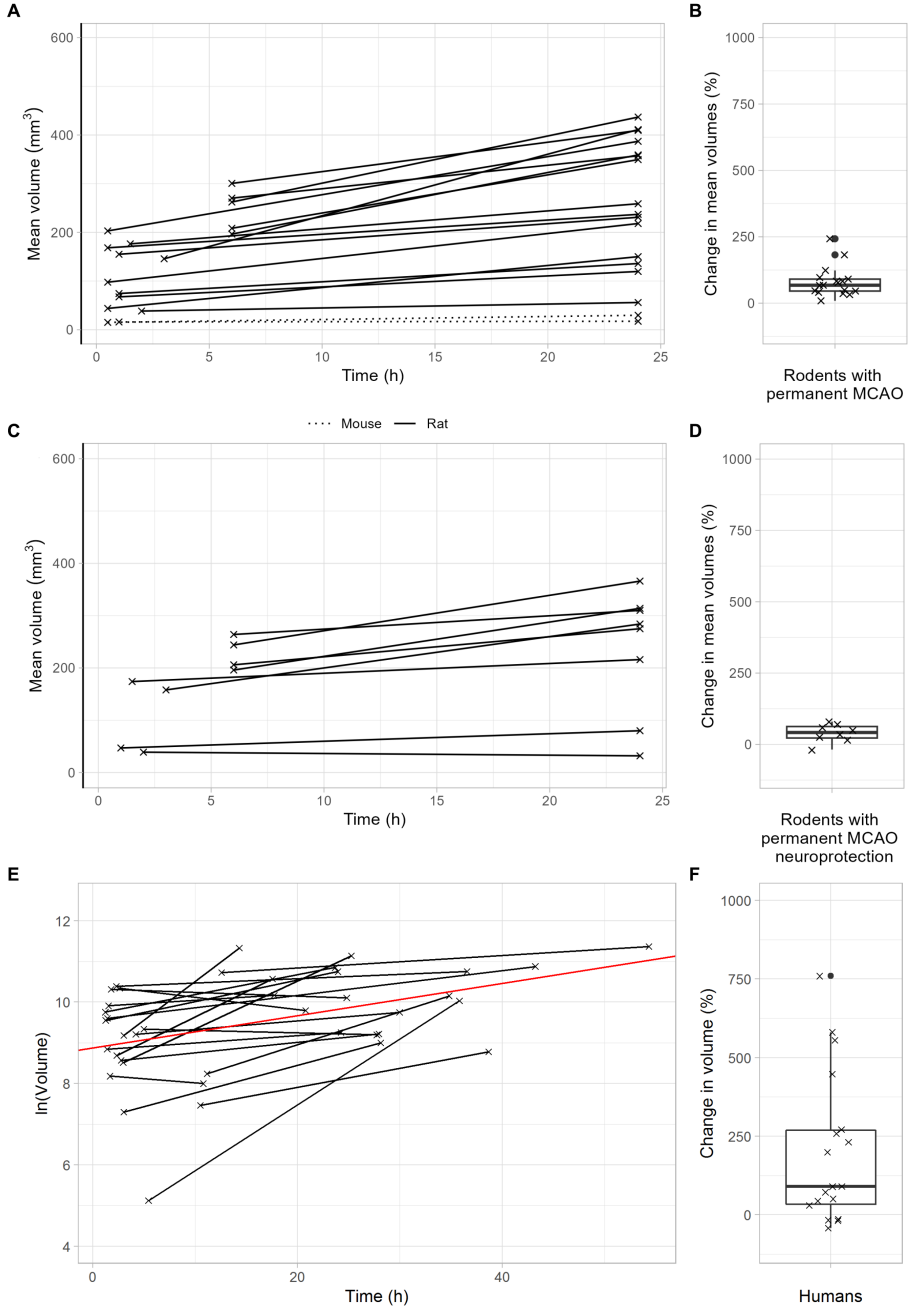
Infarct growth after permanent large vessel occlusion. **(A,B)** Infarct growth after pMCAO in rodents without neuroprotective treatment. **(A)** Black lines connect the mean infarct volumes of untreated animals determined at timepoints 1 and 2 in each individual study. **(B)** Each cross represents the change in mean infarct volumes in an individual study. The median change in mean infarct volumes was 68%. **(C,D)** Infarct growth after pMCAO in rodents with neuroprotective treatment. **(C)** Black and green lines connect the mean infarct volumes of treated animals determined at timepoints 1 and 2 in each individual study. **(D)** Each cross represents the change in mean infarct volumes in an individual study. The median change in mean infarct volumes was 42%. **(E,F)** Infarct growth in stroke patients with TICI 0-2a thrombectomy. **(E)** Black lines connect the infarct volumes at timepoints 1 and 2 in each patient. The red line represents an overall regression line given by the fixed effects of the mixed model. **(B)** Each cross represents the infarct volume growth in a patient. The median change in infarct volumes was 148%.

After permanent MCAO, rodents exhibited a median infarct volume growth of 68% (Figures 4A,B). This was reduced to 42% with neuroprotective therapy (Figures 4C,D). Importantly, stroke patients with unsuccessful recanalization (TICI 0-2a) had a meaningful infarct volume growth of 145% (Figures 4E,F). In summary, these data demonstrate that infarct volume growth depends on recanalization success and ischemia duration. Patients with either very late recanalization or unsuccessful recanalization may benefit from neuroprotective therapies.

## Discussion

Our key findings are: first, there is significant infarct volume growth after transient large vessel occlusion in animal stroke models. Second, this delayed infarct volume growth is the target of neuroprotective treatments in animal stroke. Third, most stroke patients with large vessel occlusion have no infarct growth after successful recanalization, i.e., these patients have no target for neuroprotection. Fourth, patients with either very late or unsuccessful recanalization experience meaningful infarct growth, thus offering a potential target for neuroprotection.

Our finding of negligible infarct growth in stroke patients is in line with observations from a recent multicenter clinical trial on the effect of remote ischemic perconditioning on brain infarction growth within the first 24 h after stroke onset (8). In this trial, more than 90% of patients received a recanalizing treatment, infarct volume growth was negligible (34% and 36%) and remote ischemic perconditioning did not have an additional treatment effect (8). Two other studies showed more pronounced infarct volume growth, which appears conflicting at a first glance (9, 10). However, these studies enrolled patients from 2008 until 2013 and from 2011 until 2019, respectively, so that a large proportion of patients were treated with fist-generation devices, which are associated with a higher risk of secondary injury due to thrombus fragmentation and endothelial damage (9, 10). In those patients treated with stent-retrievers, only 14% had substantial infarct volume growth (defined as infarct expansion >11.6 mL) (9).

The rapid expansion of Endovascular Therapy (EVT) facilities and the success of these interventions underscore the relevance of our findings. Our study shows minimal infarct growth in patients with successful recanalization, highlighting the narrowing window for neuroprotective strategies as EVT becomes more prevalent. This shift challenges the translation of neuroprotection from animal models to clinical practice. In light of EVT advancements, our results emphasize the urgent need to adapt neuroprotective research within this new context. Future studies should focus on optimizing neuroprotective approaches in tandem with EVT, underscoring the importance of integrating preclinical and clinical research to enhance stroke treatment efficacy.

It has to be noted that infarct volume growth might be underestimated in our cohort, because we included only patients who were amenable to receive a primary MRI scan, thus excluding unstable patients with a higher likelihood of delayed infarct growth. On the other hand, we used MRI scans upon admission as baseline scans, which, if compared to MRI scans immediately after reperfusion, may overestimate the infarct volume increase due to infarct growth between first scan and successful thrombectomy.

One limitation is that our study focused on large vessel occlusions, and our findings may not directly apply to small infarcts. The rationale for focusing on large occlusions is that the successful neuroprotection studies in rodents primarily used the tMCAO model of stroke, which corresponds to a proximal occlusion of the middle cerebral artery with successful recanalization (i.e., TICI 2b or TICI 3) in human stroke patients. By focusing on large vessel occlusions, we aim to achieve the best possible comparability between animal models and the clinical situation. We acknowledge a limitation in our comparative analysis stemming from the differing criteria for inclusion and assessment between the clinical and preclinical studies. Specifically, while the clinical studies included patients based on successful reperfusion, defined as TICI 2b-3 reperfusion, the animal studies were selected based on successful recanalization without consistent evaluation of effective reperfusion. This discrepancy may contribute to the observed differences in infarct volume growth between the animal models and clinical outcomes. The potential for less effective reperfusion in the animal models despite successful recanalization highlights a critical area for future research and underscores the necessity of rigorous reperfusion assessment in preclinical stroke models. This limitation underscores the complexity of directly comparing preclinical and clinical outcomes and emphasizes the importance of considering the nuances of reperfusion quality in translational stroke research. To better reflect the clinical situation in animal models, some other authors have proposed the establishment of large animal models of stroke. However, besides ethical concerns, there are also purely scientific doubts about whether this can truly achieve better translation, because studies with large animal models tend to have a small sample sizes and heterogeneous results, so that significant findings are difficult to obtain. Furthermore, the future implementation of preclinical multicenter studies will not be feasible with large animal models, as only a few centers with very heterogeneous research focuses have the capability to conduct large animal studies.

While the intraluminal filament model of MCAO is widely accepted and extensively used in preclinical stroke research, we acknowledge its limitations, including variability in the type of filament used and the intravascular events it induces, which may not fully replicate the complex nature of human stroke.

Previously, we identified methodological weaknesses and publication bias as major culprits for the observed efficacy decline of neuroprotective treatments from experimental studies to Phase 3 trials (4). Here, we approached this topic from another perspective and uncovered a fundamental difference in infarct evolution between rodents and humans. How can we explain such wide deviation in infarct evolution between rodents and humans? There are neuroanatomical variations in collateral systems and the proportions between gray matter and white matter (11). For instance, a complete circle of Willis is present in only 10% of C57Bl/6 J mice and Wistar rats were shown to possess particularly thin posterior communicating arteries (12, 13). Insufficient collateral blood supply and limited ability for remodeling after arterial occlusion may thus add to reduced ischemic tolerance and accelerated ischemic cell death in rodents compared to patients. Further, differences in the proportions between grey and white matter volumes may influence vulnerability to delayed ischemic cell death. Notably, the percentage of white matter accounts for 60% in humans, but decreases to 10% in mice (14). Altogether, these differences in neuroanatomical and biochemical prerequisites between species may at least partially explain unequal ischemic vulnerability.

In conclusion, our study reveals a meaningful pathophysiologic difference between animal stroke models and stroke patients: Animals have a huge infarct growth after transient middle cerebal artery occlusion (tMCAO), whereas stroke patients with large vessel occlusion do not have a meaningful infarct growth after successful thrombectomy. Assuming that infarct growth is the target of neuroprotection in animal stroke studies, most stroke patients just do not offer a target for neuroprotection after successful thrombectomy.

## Data availability statement

The raw data supporting the conclusions of this article will be made available by the authors, without undue reservation.

## Ethics statement

The studies involving humans were approved by Ethics Committee Bern (approval number: 231/149). The studies were conducted in accordance with the local legislation and institutional requirements. Written informed consent for participation was not required from the participants or the participants’ legal guardians/next of kin due to the retrospective nature of the study.

## Author contributions

AS-P: Conceptualization, Data curation, Formal analysis, Funding acquisition, Investigation, Methodology, Project administration, Resources, Software, Supervision, Validation, Visualization, Writing – original draft, Writing – review & editing. JK: Data curation, Investigation, Writing – review & editing. NB: Formal analysis, Investigation, Methodology, Writing – review & editing. NW: Investigation, Writing – review & editing. J-KS: Visualization, Writing – review & editing. MK: Investigation, Writing – review & editing. CB: Writing – review & editing. JG: Data curation, Writing – review & editing. RM: Data curation, Writing – review & editing. HW: Supervision, Writing – review & editing. HM: Conceptualization, Methodology, Supervision, Writing – review & editing. UF: Data curation, Investigation, Supervision, Writing – review & editing. JM: Conceptualization, Project administration, Resources, Supervision, Writing – review & editing.
