# Supplementary material for: The dilemma of neuroprotection trials in times of successful endovascular recanalization
Source: Front Neurol. 2024 Apr 9;15:1383494. doi: 10.3389/fneur.2024.1383494 (PMC11035835; doi:10.3389/fneur.2024.1383494)
Supplement: Supplementary file 2 [file Data_Sheet_2.PDF]

Supplemental Methods

Search strategy and selection criteria of the systematic review, and data extraction

We did a systematic review and meta-analysis according to the PRISMA and Meta-analysis of Observational Studies in Epidemiology (MOOSE) criteria. We searched PubMed using the terms: "*MRI AND stroke AND animal model AND infarct volume*", „*MRI AND stroke AND rodent AND infarct volume*", „*MRI AND stroke AND mouse AND infarct volume*" und „*MRI AND stroke AND rat AND infarct volume* " for articles published before April 10, 2020. We also hand-searched reference lists of all articles identified in the electronic search. We only selected peer-reviewed, clinical studies published in English language, including mice and rats in which MCAO had been induced and which had undergone MRI at two different time points, the first being on day one the second at least 24 hours after MCAO. For our second analysis we then, out of the previously identified ones, identified those studies that had done research for neuroprotective procedures and in which these methods had proven significantly effective. The exclusion criteria were: 1) missing report of infarct volume; (2) permanent MCAO; 3) time of MCAO not specified. To diminish systematical bias studies with fewer than 3 animals were excluded. Two researchers (ASP and NW) independently did the search, and each identified eligible studies. Any disagreements were resolved by consensus after further review of the relevant publications (ASP and NW) or, if necessary, by a third reviewer (JM). For studies published more than once (ie, duplicates), we included only the report with the most informative and complete data. We extracted data for the following characteristics: number of animals that were analysed, duration of MCAO/ischemia, infarct volume at first and second time point, infarct growth and the neuroprotective agent that was applied (if applicable).

These were then entered into two separate box plots for animals with or without applied neuroprotection.

<u>Interests, registration and protocol</u>

The authors declare no competing interests. The review was not registered, a protocol was not prepared.
