# Supplementary material for: The dilemma of neuroprotection trials in times of successful endovascular recanalization
Source: Front Neurol. 2024 Apr 9;15:1383494. doi: 10.3389/fneur.2024.1383494 (PMC11035835; doi:10.3389/fneur.2024.1383494)
Supplement: Supplementary file 3 [file Data_Sheet_3.PDF]

First Author	Year	Journal	Place of Study	MCAO Model	Occlusion time in minutes, if transient	Sex	Analysed Neuro-protectant	Time of appli-cation in (h), if neuropro-tection
Ahn	2015	Brain	Seoul, South Korea	transient, filament	90	all male rats	none	
Barber	2005	Neurosci Let	Calgary, Canada	transient, filament	30	all male mice	none	
Bardutzky	2007	Stroke	Erlangen, Germany	transient, filament	35	all male rats	none	
Baskerville	2016	JCBFM	Glasgow, UK	permanent, filament		50% female rats	none	
Campos	2011	JCBFM	Santiago de Compostela, Spain	transient, filament	90	all male rats	i.v. Glutamate oxaloacetate	1,5
Cash	2001	Brain Res	London, UK	transient, filament	120	all male rats	i.v. Aminoguanide	6
Chi	2014	Plos One	Taipei, Taiwan	transient, filament	60	all male rats	oral BNG-1 (chinese herb complex)	1
Fang	2016	Acta Neurochir	Beijing, China	transient, filament	60	all male rats	i.v. Methylene Blue	1
Gill	1996	NMR Biomed	Cambridge, UK	permanent, electrocoagulation		all male rats	i. p. MK-801 (NMDA-antagonist)	0,08
Gory	2016	Eur Rad	Bron, France	transient, filament	90	all male rats	none	
Goto	2002	Stroke	Baltimore, MD, USA	transient, filament	60	all male mice	none	
Haga	2003	Brain Res	Edinburgh, UK	transient, filament	120	all male rats	none	

Huang	2017	Stem Cell Res	San Antonio, TX, USA	transient, filament	60	all male rats	i.a. human umbilical cord blood mononuclear cells	1
Huang	2018	Brain Res	Stony Brook, NY, USA	transient, filament	60	all male rats	oral Methylene Blue	24
Jiang	2015	Plos One	Beijing, China	transient, filament	60	all male rats	none	
Kao	2017	Stroke	Chapel Hill, NC, USA	permanent, cauterization		all male mice	none	
Kim	2018	Mol Neurobiol	Yongin City, South Korea	transient, filament	120	all male rats	i.v. PARP1 Inhibitor	2
Kollmar	2002	Stroke	Heidelberg, Germany	transient, filament	120	all male rats	none	
Laigle	2013	PLOS one	Marseille, France	transient, filament	60	all male mice	none	
Liu	2006	Brain	Sapporo, Japan	permanent, filament		all male rats	i.v. human mesenchymal stem cells	3
Liu	2012	BMC Neuroscience	Kiel, Germany	transient, filament	90	all male rats	none	
Merali	2015	Transl Stroke Res	Toronto, Canada	transient, filament	60	all male rats	i.v. Imatinib	2
Moldes	2012	Neuropharmacol	Santiago de Compostela, Spain	transient, filament	90	all male rats	i.v. Clazosentan + BQ788 (Endothelin-1 antagonists)	0,5
Nagel	2008	Brain Res	Heidelberg, Germany	transient, filament	90	all male rats	i.v. Minocyclin + hypothermia	1
Nakamura	2019	J Neurosurg Sci	Sapporo, Japan	permanent, filament		all female rats	none	

Nomura	2005	Neurosci	Sapporo, Japan	permanent, filament		all male rats	i.v. human mesenchymal stem cells	6
Omori	2008	Brain Res	Sapporo, Japan	permanent, filament		all female rats	i.v. human mesenchymal stem cells	6
Onda	2008	J Cereb Blood Flow Metab	Sapporo, Japan	permanent, filament		all male rats	i.v. human mesenchymal stem cells	6
Petty	2003	Eur J Pharmacol	Bridgewater, NJ, USA	transient, filament	120	all male rats	i.v. ACEA 1021 (NMDA antagonist)	0,5
Pialat	2008	NMR Biomed	Lyon, France	permanent, electrocoagulation		all male mice	none	
Reid	2012	JCBFM	Glasgow, UK	permanent, filament		all male rats	none	
Robertson	2015	Int J Stroke	Glasgow, UK	permanent, filament		all male rats	none	
Rodriguez	2014	Brain Res	San Antonio, Tx, USA	permanent, filament		all male rats	none	
Rodriguez	2016	Brain Behav	San Antonio, Tx, USA	transient, filament	60	all male rats	i.v. Methylene Blue + Normobaric Oxygenation	0,5
Rudin	2001	Exp Neurol	Basel, Switzerland	permanent, electrocoagulation		all male rats	none	
Schäbitz	2004	Stroke	Heidelberg, Germany	permanent, filament		all male rats	Hyperbaric oxygenation	2

Schäbitz	2001	Stroke	Heidelberg, Germany	transient, filament	60	all male rats	intraventricular IGF-1	0,5
Schatlo	2008	JCBFM	Bethesda, MD, USA	transient, filament	360	all male rats	none	
Shen	2013	Plos One	San Antonio, TX, USA	transient, filament	60	all male rats	i.v. MB	1
Sironi	2003	Arterioscler Thromb Vasc Biol	Milano, Italy	permanent, electrocoagulation		all male rats	s.c. Simvastatin	3
Son	2015	J Neurochem	Seoul, South Korea	transient, filament	120	all male rats	none	
Taheri	2014	Brain Res	Charleston, SC, USA	transient, filament	90	all male rats	none	
Tian	2009	Neurosci Lett	Shanghai, China	transient, filament	120	all male rats	none	
Ukai	2007	J Neurotrauma	Sapporo, Japan	permanent, filament		all female rats	i.v. human mesenchymal stem cells	6
Veltkamp	2005	Brain Res	Heidelberg, Germany	transient, filament	120	all male rats	none	
Vieites-Prado	2016	Stroke	Santiago de Compostela, Spain	transient, filament	45	all male rats	Systemic/focal Hypothermia	1
Walberer	2008	J Neurosurg Sci	Giessen, Germany	transient, filament	90	all male rats	none	
Wang	2007	Neuropharm	Jiangsu, China	transient, filament	120	all male rats	none	
Wei	2003	JCBFM	Galveston, TX, USA	transient, filament	90	all male rats	i.v. 2-Deoxy-D-Glucose	0,17
Woo	2017	Plos One	Seoul, South Korea	transient, filament	60	all male rats	none	
